# Validation of the German basic psychological need satisfaction in sleep scale

**DOI:** 10.3389/fpsyg.2025.1611551

**Published:** 2025-09-17

**Authors:** Patricia Frytz, Lucie Nikoleizig, Anne-Marie Elbe

**Affiliations:** ^1^Department of Sport Psychology, Sport Science Faculty, Leipzig University, Leipzig, Germany; ^2^Laboratory for Sleep, Cognition and Consciousness Research, Department of Psychology, University of Salzburg, Salzburg, Austria; ^3^Faculty of Culture, Media & Psychology, Macromedia University of Applied Science, Leipzig, Germany

**Keywords:** questionnaire, sleep quality, basic needs, well-being, self-determination theory

## Abstract

Basic psychological need theory posits that satisfying basic psychological needs (autonomy, competence, relatedness) is connected to overall well-being and physiological health, including sleep. The reciprocal relationship between basic psychological need satisfaction and sleep quality has been investigated in different studies. However, a domain-specific questionnaire exploring basic psychological need satisfaction with a focus on sleep does not exist. Therefore, in study 1 we constructed the Basic Psychological Need Satisfaction in Sleep Scale (BPNSS-S) through confirmatory factor analysis and item selection. A total of 227 participants completed the first version of the questionnaire. In study 2, 295 participants answered the final version of the 7-item questionnaire including measurements on sleep quality, life satisfaction, vigorous exercise and basi psychological need satisfaction in exercise. The final BPNSS-S yielded good to excellent model fit, and agreement with other scales supports the assumptions of good criterion, construct, and discriminant validity. The newly developed BPNSS-S is a reliable and valid tool for assessing basic psychological need satisfaction in the sleep domain. Application of the scale will help researchers and practitioners better understand and improve sleep quality and well-being through the lens of need satisfaction. This study lays the foundation for further studies in sleep and needs research, with the potential to guide targeted approaches that enhance sleep behavior across various populations.

## 1 Introduction

When we mention “needs” in everyday life, we are usually referring to what we need to maintain our basic bodily functions and sustain life.

Sleep is one of the basic physiological needs and plays a crucial yet frequently underappreciated role in overall physical and mental health and functioning. Restful sleep is vital for the body’s restorative processes including physical recovery and immune and cognitive functioning (Assefa et al., 2015). Chronic sleep deprivation can lead to severe physical and mental health consequences, including impaired psychological well-being, and increased health-risk behaviors and risk of chronic diseases such as cardiovascular disease or diabetes (Jike et al., 2018; Dai et al., 2020). Despite its importance, many individuals face obstacles in achieving sufficient, high-quality sleep. According to the Phillips Global Sleep Survey (2019), up to 67% of adults worldwide reported at least one sleep disruption per night. High sleep quality can be defined via various parameters. Objective factors include sufficient sleep duration relative to age group, high sleep efficiency, short sleep onset latency, and low incidence of wake after sleep onset (WASO; Watson et al., 2015; Ohayon et al., 2017). The subjective experience of high sleep quality can be defined in terms of basic needs: ‘sleep need’ refers to the fulfillment of individual sleep requirements (Sargent et al., 2021).

In addition to basic physiological needs, psychological needs ensure individual well-being, optimal functioning, and personal growth, as outlined in self-determination theory (SDT; Ryan and Deci, 2000). One of six central mini-theories of SDT, the basic psychological need theory (BPNT; Ryan and Deci, 2017), defines psychological needs as innate, universal, distinguishable from other needs, and essential for psychological functioning and adjustment, with their frustration linked to problematic behavior, ill-being, and psychopathology (Vansteenkiste et al., 2020). BPNT identifies three such needs: autonomy, competence, and relatedness. *Autonomy* denotes the experience of volition and self-endorsement in one’s actions; *competence* refers to feeling effective and capable of achieving desired outcomes; and *relatedness* refers to the need to feel connected, cared for, and a sense of belonging. Evidence has shown that while these three needs are highly correlated, they are distinct (Rackow et al., 2013; Neubauer and Voss, 2016). Within BPNT, Ryan and Deci (2017) attribute the satisfaction of psychological needs to wellness, which is defined as *thriving* or *fully functioning* rather than happiness or the presence/absence of positive/negative emotions. Need frustration, conversely, is associated to ill-being and impoverished functioning. Key components of *thriving* include vitality, awareness, access to, and application of one’s individual capacities and authentic self-regulation. *Fully functioning* reflects the individual’s ability to be open to new experiences, being reflective and to integrate inner and outer outputs (inner needs and states) into coherent behavior (Perls et al., 1951). It is stated that the satisfaction of basic needs buffers and mediates the effects of adverse life circumstances on wellness, vitality, and motivation. The satisfaction of autonomy, competence and relatedness needs, individually and interactively, appears crucial to overall well-being across ages, contexts, and cultures, not only situationally but also developmentally (Ryan et al., 2010; Lataster et al., 2022; Soenens and Vansteenkiste, 2023). Additionally, recent studies found need satisfaction to serve as a protective factor on individual’s well-being in adverse life situations like the COVID-19 pandemic (Horvát et al., 2025; Kiltz et al., 2024). The relationship between need satisfaction and well-being within the BPNT framework has also been detected to work in specific life domains, for instance in traveling, sports or the classroom (Çiki and Tanriverdİ, 2025; Jiang and Shen, 2025; Shelton-Strong, 2025).

There is evidence that basic psychological need satisfaction has an effect on physiological health, including for example, cholesterol levels, cortisol secretion, and body mass index (Quested et al., 2011; Uysal et al., 2020). This research indicates that there may be a link between basic psychological needs and physiological outcomes and needs, among others. It has been shown that the satisfaction or frustration of psychological needs is linked to eating regulation (Verstuyf et al., 2013), sexual interactions (Smith, 2007), and physical safety (Chen et al., 2015a). There are also studies indicating a relation between psychological needs and sleep. Subjective sleep quality has been found to be enhanced by need satisfaction both in the short and long term, even revealing a predictive nature of needs (Campbell et al., 2015; Uysal et al., 2020). In contrast, evidence on daily need satisfaction revealed a reciprocal relationship pointing to a dynamic interplay between the two constructs (Campbell et al., 2018). Furthermore, those studies were conducted in clinical and non-clinical populations from different age groups. For instance, a study of older individuals in China describes longitudinal as well as lagged interplays between need satisfaction and sleep with an emphasis on relatedness (Li, 2023). Similar results were found in a cohort of adolescents (Campbell et al., 2021) and HIV-patients (Campbell et al., 2019). Besides that, subjective energy levels, symptoms of stress, and arousal processes are considered mediating effects of this relationship (Campbell and Vansteenkiste, 2023). Taken together, these findings highlight that the link between psychological need satisfaction and sleep quality is dynamic, reciprocal, and observable across populations and time scales, with growing attention to the processes that may explain how and why this relationship unfolds.

The above-mentioned studies focus on general need satisfaction in daytime activities and situations, using domain-general instruments like the Balanced Measure of Psychological Needs (Sheldon and Hilpert, 2012) or the Basic Psychological Need Satisfaction and Need Frustration Scale (Chen et al., 2015b). To date, there is no research on sleep-specific basic psychological need satisfaction. This is in stark contrast to other areas of life where domain-specific instruments on need satisfaction do exist. Examples are the Basic Psychological Need Satisfaction Scale—Relationship Domain (La Guardia et al., 2000) or the Basic Psychological Need Satisfaction Scale—Work Domain (Kasser et al., 1992; Ilardi et al., 1993; Deci et al., 2001). The German Psychological Need Satisfaction in Exercise Scale (PNSEG; Rackow et al., 2013) focuses on need satisfaction during physical activity and the Basic Psychological Need Satisfaction in Active Commuting to and from School (Burgueño et al., 2020) has an even narrower focus. Beyond these, there are very few scales that measure domain-specific basic psychological need satisfaction in the German language (Grünwald et al., 2024). This paucity of questionnaires highlights the need for German, domain-specific instruments that adequately assess the psychological model of needs (Ryan, 1995; Vlachopoulos and Michailidou, 2006).

Based on the previous studies regarding the relationship between need satisfaction and sleep, we hypothesize that improving need fulfillment specifically within the realm of sleep-related behaviors may significantly enhance sleep quality. To achieve this, it is essential to utilize appropriate instruments that can accurately assess an individual’s sleep related need satisfaction. Only then can suitable interventions be deducted. Thus, the aim of this research is to develop a domain-specific questionnaire of basic psychological need satisfaction for sleep quality based on the three basic psychological needs of competence, autonomy and relatedness. Such a domain-specific tool could help to enhance individual’s sleep quality and overall well-being and could shed light on the interrelations between sleep and basic psychological need satisfaction. Further, it enables us to deduce suitable interventions. To date, such an instrument in the sleep domain does not exist. While sleep itself is a state with rather reduced consciousness and responsiveness (Windt, 2020), we interpret sleep in this study as a life domain. This is outlined as a specific area of an individual’s life, e.g., work or family, that contribute to overall well-being (Sirgy, 2021). Therefore, we used a broad approach to sleep behavior that also includes actions and practices that are related to sleep like sleep routines or planning sleep schedules.

In order to develop a domain-specific instrument, we conducted two studies. In study 1 the scale was developed and its factorial validity was analyzed. Study 2 focused on determining criterion, construct, and discriminant validity for the final version of the questionnaire. In line with the foundations of self-determination theory (SDT; Ryan and Deci, 2000) we expected that sleep-specific need satisfaction would be positively associated with better sleep quality and higher overall life satisfaction (Uysal et al., 2020; Campbell et al., 2021). We also assumed that our scale would show correlations with need satisfaction in other life domains. We chose to examine psychological need satisfaction in the physical exercise context, as physical activity and sleep are both aspects of physiological health (Luyster et al., 2012; Lazarus et al., 2019) and therefore represent related life domains. As a distinguishing feature between these two domains we used vigorous exercise as a parameter (Rackow et al., 2013). We expected a positive correlation between vigorous exercise and need satisfaction in exercise, but not between vigorous exercise and need satisfaction in sleep.

## 2 Study 1: Questionnaire development and factorial validation

### 2.1 Method

#### 2.1.1 Sample and procedure

Data were collected between December 2023 and July 2024 using an online questionnaire administered through LimeSurvey (LimeSurvey, 2023). The study was conducted in accordance with the Declaration of Helsinki and ethical guidelines of the American Psychological Association (APA). Participation was voluntary and informed consent was obtained from all participants prior to filling out the questionnaires. Participants were informed that study consent could be withdrawn at any time and that their data would be deleted.

Participants were recruited by distributing the survey in different lectures for bachelor and master students [blinded] and in sleep workshops for athletes.

The sample consisted of 227 participants with mean (*M*_Age_) and standard deviation (*SD*) of *M*_Age_ = 22.71, *SD*_Age_ = 4.66 and 59.0% females. Three participants did not indicate their age or gender. The sample size met the power requirements of 20 participants per item (Schumacker and Lomax, 2015). The majority of participants were students (55.5%) and were physically active in regional competitive sport (46.7%) followed by recreational sport (42.3%). No participants were excluded.

#### 2.1.2 Measures

All participants completed the first version of the BPNSS-S and provided demographic information.

##### 2.1.2.1 The German basic psychological need satisfaction in sleep scale

The preliminary version of the BPNSS-S (Pre-BPNSS-S) consisted of 11 items, and was guided by existing questionnaires on domain-specific basic need satisfaction. Items were developed by the first author as well as critically discussed, evaluated, and updated within the author team. The initial version consisted of 3 items for the subscale autonomy (e.g., “I feel like I can decide for myself how long I sleep”) and 4 items for each of the subscales competence (e.g., “I feel like I have effective strategies for getting enough sleep/recovery”) and relatedness (e.g., “There are people I can trust when I talk about sleep problems.”). Participants rated the items on a 7-point Likert scale ranging from 1 (*do not agree at all*) to 7 (*very strongly agree*).

##### 2.1.2.2 Demographics

Demographic information such as age (in years), gender (female, male, non-binary, other), and highest educational qualification (e.g., high school diploma, bachelor’s or master’s degree) were collected to describe sample characteristics. Additionally, data on current employment status (e.g., student, employee, pensioner) and on current physical activity (e.g., competitive sport, recreational sport, no physical activity) were gathered.

#### 2.1.3 Data analysis

We used SPSS 29 to measure descriptive statistics, scale values, reliability analyses, and correlations between the scales. Due to violations of the normal distribution we applied Spearman correlations. Model fit, including confirmatory factor analysis (CFA) and item selection, was conducted using RStudio 2024 using psych, ltm, readxl, and lavaan packages.

We first checked the Pre-BPNSS-S for descriptive statistics (skewness, kurtosis, item-rest correlation), factor loadings, internal consistency, and intercorrelations. Kurtosis and skewness were used to determine item distribution. Psychometric reliability was reported through item-rest correlation and factor loadings. We then evaluated the 3-factor structure (autonomy, competence, relatedness) of the questionnaire using CFA. We assessed several goodness-of-fit indices to determine model fit, including the chi-square statistic (χ^2^), the comparative fit index (CFI), the Tucker-Lewis index (TLI), the standardized root mean square residual (SRMR), the root mean square error of approximation (RMSEA), the goodness-of-fit index (GFI), and the adjusted GFI (AGFI). We then checked for possible improvements in reliability and selectivity measures through item selection and evaluated the respective parameters (descriptive statistics, CFA) of the final questionnaire version (BPNSS-S).

### 2.2 Results

#### 2.2.1 Descriptive statistics, correlations and factor loadings for the subscales of the pre-BPNSS-S

**Figure 1:**
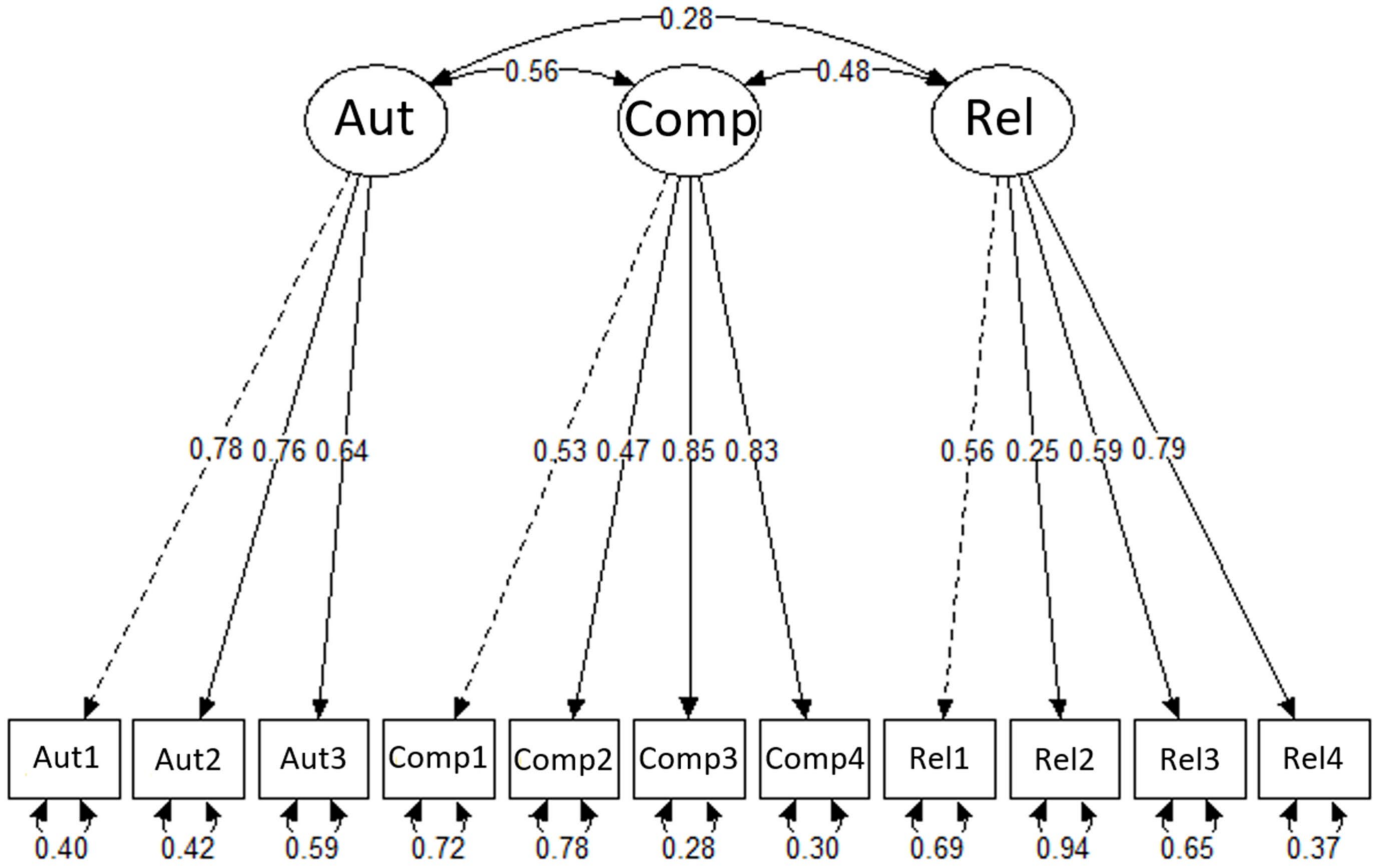
Pre-BPNSS-S measurement model showing relationships between the three subscales autonomy (Aut), competence (Comp) and relatedness (Rel). Each subscale is measured by its respective items (shown as boxes). Standardized factor loadings are displayed along the one-headed paths connecting subscales and items. The double-headed arrows represent the covariance of correlation between the subscales (top) and the residuals for each item (bottom).

Participants yielded average item scores of 4.47 (*SD* = 1.32) for autonomy, 3.98 (*SD* = 1.23) for competence, and 4.69 (*SD* = 1.17) for relatedness in the Pre-BPNSS-S with regard to their sleep (see Table 1). Skewness and kurtosis values implied that the scores tended to deviate from normality. Most items were negatively skewed. However, score values did not significantly exceed normality for any one item (skewness < 2; kurtosis < 7; West et al., 1995). Item-rest correlations (corrected item-total correlations) were weak to acceptable for the subscale relatedness (*r*_**ir**_ = 0.28–0.53), acceptable to strong for the subscale competence (*r_ir_* = 0.44–0.70) and strong for autonomy (*r_ir_* = 0.51–0.64; Field, 2009). The items revealed low (*λ* = 0.25) to strong (*λ* = 0.85) factor loadings. Communalities ranged between weak and high values with 7 to 72% (Rel2 – Comp3) of the variance of each item explained by the underlying factor (see Table 2). Item residuals with values from 0.28 to 0.94 indicated low to high error variance (see Figure 1).

**Table 1:** Descriptive statistics for items of the preliminary vesion of the German basic psychological need satisfaction in sleep scale (Pre-BPNSS-S).

Items	*M*	*SD*	Skewness	Kurtosis	r_ir_
Autonomy (Cronbach’s α: 3 items = 0.76)					
**Aut1:** Ich habe das Gefühl, selbst entscheiden zu können, wie lange ich schlafe. [I feel like I can decide for myself how long I sleep.]	4.40	1.68	−0.23	−0.85	0.64
**Aut2:** Ich habe das Gefühl, selbst entscheiden zu können, wann ich ins Bett gehe und wann ich aufstehe. [I feel like I can decide for myself when I go to bed and when I get up.]	4.37	1.64	−0.23	−0.80	0.64
**Aut3:** Ich habe das Gefühl, dass ich für meine Erholung im Schlaf das machen kann, was mir gut tut. [I have the feeling that I can do what is good for my night-time recovery.]	4.63	1.48	−0.31	−0.38	0.51
Competence (Cronbach’s α: 4 items = 0.76)					
**Comp1:** Ich kenne gute Strategien, um mit Schlafproblemen umzugehen. [I know good strategies to deal with sleep problems.]	3.63	1.67	0.36	−0.69	0.50
**Comp2:** Ich kann Phasen, in denen ich schlecht oder zu wenig schlafe, gut bewältigen. [I can cope well with phases in which I sleep badly or too little.]	4.22	1.62	−0.08	−0.88	0.44
**Comp3:** Ich habe den Eindruck, dass ich effektive Strategien habe, um mir genug Schlaf/Erholung zu holen. [I have the impression that I have effective strategies for getting enough sleep/recovery.]	3.88	1.56	0.09	−0.64	0.70
**Comp4:** Ich bin auf einem guten Weg, meine Schlafziele zu erreichen. [I am well on my way to achieving my sleep goals.]	4.20	1.57	−0.22	−0.64	0.64
Relatedness (Cronbach’s α: 4 items = 0.63)					
**Rel1:** Ich fühle mich mit den Personen verbunden, mit denen ich rede, wenn es um meine Schlaf-/Regenerationszeit geht. [I feel connected to the people I talk to when it comes to my sleep/recovery time.]	4.39	1.61	−0.31	−0.41	0.42
**Rel2:** Es gibt Menschen, die sich um meinen Schlaf und meine Erholungszeit sorgen. [There are people who worry about my sleep and recovery time.]	3.79	1.98	0.05	−1.20	0.28
**Rel3:** Es gibt Menschen, denen ich vertrauen kann, wenn ich über Schlafprobleme spreche. [There are people I can trust when I talk about sleep problems.]	5.74	1.56	−1.41	1.39	0.53
**Rel4:** Ich fühle mich sehr wohl, wenn ich mit anderen über meine Schlafroutinen rede. [I feel very comfortable talking to others about my sleep routines.]	4.83	1.60	−0.38	−0.46	0.46

r_ir_ = Item-rest correlation (corrected item-total correlation). *N* = 227.

**Table 2:** Parameter estimates (factor loadings, communalities) for the Pre-BPNSS-S.

Item	Need factors			*R^2^*
	Autonomy	Competence	Relatedness	
**Aut1:** Ich habe das Gefühl, selbst entscheiden zu können, wie lange ich schlafe. [I feel like I can decide for myself how long I sleep.]	0.78			0.60
**Aut2:** Ich habe das Gefühl, selbst entscheiden zu können, wann ich ins Bett gehe und wann ich aufstehe. [I feel like I can decide for myself when I go to bed and when I get up.]	0.76			0.58
**Aut3:** Ich habe das Gefühl, dass ich für meine Erholung im Schlaf das machen kann, was mir gut tut. [I have the feeling that I can do what is good for my night-time recovery.]	0.64			0.41
**Comp1:** Ich kenne gute Strategien, um mit Schlafproblemen umzugehen. [I know good strategies to deal with sleep problems.]		0.53		0.28
**Comp2:** Ich kann Phasen, in denen ich schlecht oder zu wenig schlafe, gut bewältigen. [I can cope well with phases in which I sleep badly or too little.]		0.47		0.22
**Comp3:** Ich habe den Eindruck, dass ich effektive Strategien habe, um mir genug Schlaf/Erholung zu holen. [I have the impression that I have effective strategies for getting enough sleep/recovery.]		0.85		0.72
**Comp4:** Ich bin auf einem guten Weg, meine Schlafziele zu erreichen. [I am well on my way to achieving my sleep goals.]		0.84		0.70
**Rel1:** Ich fühle mich mit den Personen verbunden, mit denen ich rede, wenn es um meine Schlaf-/Regenerationszeit geht. [I feel connected to the people I talk to when it comes to my sleep/recovery time.]			0.56	0.31
**Rel2:** Es gibt Menschen, die sich um meinen Schlaf und meine Erholungszeit sorgen. [There are people who worry about my sleep and recovery time.]			0.25	0.07
**Rel3:** Es gibt Menschen, denen ich vertrauen kann, wenn ich über Schlafprobleme spreche. [There are people I can trust when I talk about sleep problems.]			0.59	0.35
**Rel4:** Ich fühle mich sehr wohl, wenn ich mit anderen über meine Schlafroutinen rede. [I feel very comfortable talking to others about my sleep routines.]			0.79	0.63

*N* = 227.

The subscales were correlated as follows: autonomy and competence: *r*(225) = 0.41, *p* < 0.001; competence and relatedness: *r*(225) = 0.30, *p* < 0.001; autonomy and relatedness: *r*(225) = 0.12, *p* = 0.069.

#### 2.2.2 Confirmatory factor analysis and item selection

We then tested the factor structure of the Pre-BPNSS-S via CFA. The χ^2^-test was significant, indicating imperfect model fit. The normed value (χ^2^/*df*) showed an acceptable fit (< 3; Schermelleh-Engel et al., 2003). CFI value was above 0.90 suggesting a good fit. TLI slightly below 0.90 seemed acceptable, but not ideal (Hu and Bentler, 1999). Using a threshold of 0.08, the SRMR (0.070) and RMSEA (0.079) pointed to acceptable to good fit (Browne and Cudeck, 1993; Hu and Bentler, 1999). While the GFI showed good model fit with a value >0.90, the AGFI indicated reasonable fit with a value slightly <0.90 (Hooper et al., 2008). See Table 3 for a summary of the fit indices. Reliability analyses showed a Cronbach’s *α* ranging from 0.63 (subscale relatedness) to 0.76 (subscales autonomy, competence), indicating questionable to adequate internal consistency.

**Table 3:** Chi-square values and fit indices of the tested models.

Model	χ^2^	*df*	χ^2^/*df*	CFI	TLI	SRMR	RMSEA	GFI	AGFI
Pre-BPNSS-S	99.06**	41	2.42	.917^a^	.889^a^	.070^a^	.079^a^	.925^g^	.879^n^
BPNSS-S	17.25	11	1.57	.986^g^	.974^g^	.029^a^	.05^g^	.979^g^	.946^g^

χ^2^, chi-square; df, degrees of freedom; χ^2^/df, normed chi-square; CFI, comparative fit index; TLI, Tucker-Lewis index; SRMR, standardized root mean square residual; RMSEA, 90% confidence interval of the root mean square error of approximation; GFI, goodness-of-fit index; AGFI, adjusted goodness-of-fit index. g, good value; a, acceptable value; n, unacceptable value. **p < 0.001.

Low psychometric reliability and low internal consistency as well as several almost acceptable goodness-of-fit indices indicated that further improvements were needed, including deleting items from the initial version of the scale. We thus did the following.

Items were deleted stepwise from the original model to check for improvements in reliability and selectivity measures. In the first step, the deletion of items Aut3, Comp2 and Rel2 from the questionnaire improved the reliability of the subscales, as indicated by item-deletion reliability analysis. Scale reliability (0.77) further improved after deleting Comp1 in a second step. No further improvement in internal consistency could be achieved by further item deletion, indicating a final 7-item model. In the resulting BPNSS-S, Cronbach’s α improved for all scales ranging from 0.67 (subscale relatedness) to 0.82 (subscale competence), showing satisfactory to good internal consistency (see Table 4; Taber, 2018). Compared to the Pre-BPNSS-S, all goodness-of-fit indices yielded optimized fit (see Table 3 for model comparison). The non-significance of the χ^2^-test including a χ^2^/*df* ratio <2 indicated good model fit and showed that the model’s complexity was well-justified by the data (Schermelleh-Engel et al., 2003). CFI and TLI values, adjusted for model complexity, showed values >0.95 signaling excellent model fit (Hu and Bentler, 1999). An SRMR value far below 0.08 pointed to very good model fit. With a value at the threshold for good fit (0.05), the RMSEA suggested that residuals in the tested model were small (Browne and Cudeck, 1993; Hu and Bentler, 1999). Very good to excellent model fit was indicated by a GFI > 0.95 and excellent fit by an AGFI close to the threshold (0.946; Hooper et al., 2008). In summary, the goodness-of-fit indices with good to excellent values indicated a strong fit between the proposed model and the observed data.

**Table 4:** Descriptive statistics for BPNSS-S items.

Items	*M*	*SD*	Skewness	Kurtosis	*r*_ir_
Autonomy (Cronbach’s α: 2 items = 0.77)					
**Aut1:** Ich habe das Gefühl, selbst entscheiden zu können, wie lange ich schlafe. [I feel like I can decide for myself how long I sleep.]	4.40	1.68	−0.23	−0.85	0.63
**Aut2:** Ich habe das Gefühl, selbst entscheiden zu können, wann ich ins Bett gehe und wann ich aufstehe. [I feel like I can decide for myself when I go to bed and when I get up.]	4.37	1.64	−0.23	−0.80	0.63
Competence (Cronbach’s α: 2 items = 0.82)					
**Comp3:** Ich habe den Eindruck, dass ich effektive Strategien habe, um mir genug Schlaf/Erholung zu holen. [I have the impression that I have effective strategies for getting enough sleep/recovery.]	3.88	1.56	0.09	−0.64	0.70
**Comp4:** Ich bin auf einem guten Weg, meine Schlafziele zu erreichen. [I am well on my way to achieving my sleep goals.]	4.20	1.57	−0.22	−0.64	0.70
Relatedness (Cronbach’s α: 3 items = 0.67)					
**Rel1:** Ich fühle mich mit den Personen verbunden, mit denen ich rede, wenn es um meine Schlaf-/Regenerationszeit geht. [I feel connected to the people I talk to when it comes to my sleep/recovery time.]	4.39	1.61	−0.31	−0.41	0.45
**Rel3:** Es gibt Menschen, denen ich vertrauen kann, wenn ich über Schlafprobleme spreche. [There are people I can trust when I talk about sleep problems.]	5.74	1.56	−1.41	1.39	0.46
**Rel4:** Ich fühle mich sehr wohl, wenn ich mit anderen über meine Schlafroutinen rede. [I feel very comfortable talking to others about my sleep routines.]	4.83	1.60	−0.38	−0.46	0.55

r_ir_ = Item-rest correlation (corrected item-total correlation). *N* = 227.

#### 2.2.3 Descriptive statistics, correlations and factor loadings for the three final subscales

Regarding sleep, average item scores of the final version of the BPNSS-S were 4.39 (*SD* = 1.50) for autonomy, 4.04 (*SD* = 1.44) for competence, and 4.99 (*SD* = 1.24) for relatedness (see Table 4). These results were also reflected in skewness and kurtosis of the individual items. Both values indicated that the results tended to deviate from normality and were negatively skewed. Nevertheless, the values did not deviate substantially from normality (skewness < 2; kurtosis < 7) for any one item (West et al., 1995). Item-rest correlations (corrected item-total correlations) were in an acceptable range for the subscale relatedness (*r_ir_* = 0.45–0.55) and showed strong values for the subscales autonomy (*r_ir_* = 0.63) and competence (*r_ir_* = 0.70; Field, 2009). Factor loadings of the items were acceptable (*λ* = 0.54) to strong (*λ* = 0.82). From 29 to 77% (Rel1 – Comp4) of the variance was explained by its underlying factor (see Table 5), pointing to communalities in the range between the lower boundary of moderate values and high values. Item residuals with values from 0.23 to 0.71 indicated low to high error variance (see Table 5).

**Table 5:** Parameter estimates for the BPNSS-S.

Item	Need factors			*R^2^*
	Autonomy	Competence	Relatedness	
**Aut1:** Ich habe das Gefühl, selbst entscheiden zu können, wie lange ich schlafe. [I feel like I can decide for myself how long I sleep.]	0.82			0.68
**Aut2:** Ich habe das Gefühl, selbst entscheiden zu können, wann ich ins Bett gehe und wann ich aufstehe. [I feel like I can decide for myself when I go to bed and when I get up.]	0.76			0.58
**Comp3:** Ich habe den Eindruck, dass ich effektive Strategien habe, um mir genug Schlaf/Erholung zu holen. [I have the impression that I have effective strategies for getting enough sleep/recovery.]		0.80		0.63
**Comp4:** Ich bin auf einem guten Weg, meine Schlafziele zu erreichen. [I am well on my way to achieving my sleep goals.]		0.88		0.77
**Rel1:** Ich fühle mich mit den Personen verbunden, mit denen ich rede, wenn es um meine Schlaf-/Regenerationszeit geht. [I feel connected to the people I talk to when it comes to my sleep/recovery time.]			0.54	0.29
**Rel3:** Es gibt Menschen, denen ich vertrauen kann, wenn ich über Schlafprobleme spreche. [There are people I can trust when I talk about sleep problems.]			0.54	0.30
**Rel4:** Ich fühle mich sehr wohl, wenn ich mit anderen über meine Schlafroutinen rede. [I feel very comfortable talking to others about my sleep routines.]			0.84	0.71

*N* = 227.

**Figure 2:**
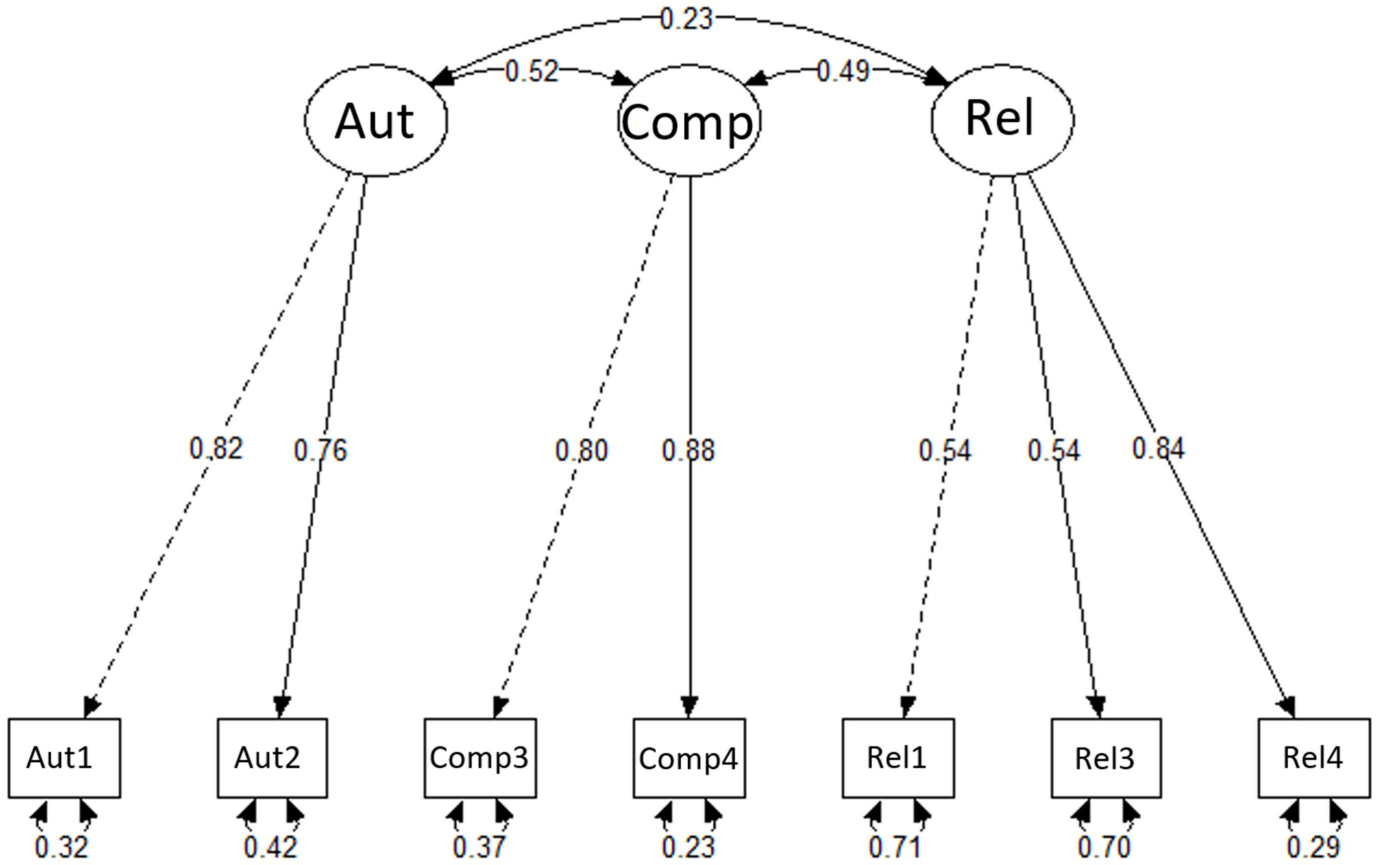
BPNSS-S measurement model showing relationships between the three subscales autonomy (Aut), competence (Comp), and relatedness (Rel). Each subscale is measured by its respective items (boxes). Standardized factor loadings are displayed along the one-headed paths connecting subscales and items. The double-headed arrows represent the covariance of correlation between the subscales (top) and the residuals for each item (bottom).

The three basic needs were positively correlated to each other: autonomy and competence: *r*(225) = 0.40, *p* < 0.001; competence and relatedness: *r*(225) = 0.38, *p* < 0.001; autonomy and relatedness: *r*(225) = 0.16, *p* = 0.015. Figure 2 depicts the final measurement model (BPNSS-S).

## 3 Study 2: Questionnaire validation

Using the BPNSS-S developed in study 1, we conducted a second study to determine content validity (criterion, construct, discriminant) of the scale. As described in the introduction, we expected the scale to correlate with related constructs of sleep quality and life satisfaction within the framework of the BPNT. We also expected to find a relationship of the scale to need satisfaction in other life domains. Due to its shared association with physiological health, we chose the domain of physical exercise. We further chose vigorous activity measured by the IPAQ (Booth, 2000) as a parameter to show that the sleep domain addressed in the BPNSS-S can be distinguished from the exercise domain, as the latter has been used as criterion validity parameter in the validation study for the German PNSEG (Rackow et al., 2013) with the aim to develop a need satisfaction scale for the sport domain.

### 3.1 Method

#### 3.1.1 Sample and procedure

The survey was distributed online to bachelor students [blinded], personal contacts and through social media platforms. The study was conducted in accordance with the Declaration of Helsinki and ethical guidelines of the American Psychological Association (APA). Participation was voluntary and informed consent was obtained from all participants prior to filling out the questionnaires. Study consent could be withdrawn at any time and data deleted.

A total of 295 participants (*M*_Age_ = 29.12, *SD*_Age_ = 11.43; 62.7% females; 2 diverse individuals) were recruited for study 2. Students comprised 61.7% (*n* = 182) of the participants and 31.5% (*n* = 93) were employees. Of the total, 68.5% (*n* = 202) were active in recreational sport, 5.4% (*n* = 16) in national sport, 8.5% (*n* = 25) in regional competitive sport, 13.6% (*n* = 40) were not active in sport, and 4.1% (*n* = 12) stated ‘other’. Six participants were excluded from the data analysis due to missing data.

#### 3.1.2 Measures

Participants filled in the BPNSS-S, the Pittsburgh Sleep Quality Index (PSQI; Buysse et al., 1989), the German Version of the Satisfaction with Life Scale (SWLS; Glaesmer et al., 2011), the PNSEG (Rackow et al., 2013), the International Physical Activity Questionnaire (IPAQ; Booth, 2000) and provided demographic data.

##### 3.1.2.1 The Pittsburgh sleep quality index

The PSQI (Buysse et al., 1989) served as an indicator for sleep quality to check for criterion validity. It is the most commonly used instrument in this field (Mollayeva et al., 2016) and measures sleep quality and disturbance during the past 4 weeks via 19 items ranging from 0 (*no difficulties*) to 3 (*great difficulties*). Taken together, the seven components (sleep duration, sleep disturbance, sleep latency, daytime dysfunction due to sleepiness, sleep efficiency, overall sleep quality, and sleep medication use) yield a sum score of 0–21. Participants with a score of 0–5 are defined as good sleepers, whereas scores of 6–10 or >10 indicate significant sleep disturbances or chronic sleep disorders, respectively. Reliability in the present sample featured a Cronbach’s *α* = 0.64.

##### 3.1.2.2 The German version of the satisfaction with life scale

As previously used in similar validation studies to examine criterion validity of need satisfaction scales (Neubauer and Voss, 2016; Heissel et al., 2019), we used the German Version (Glaesmer et al., 2011) of the SWLS (Diener et al., 1985) to assess well-being of the participants. The scale consists of five items (e.g., “I am satisfied with my life”) and participants answered the items on a 7-point Likert scale ranging from 1 (*strongly disagree*) to 7 (*strongly agree*). Internal consistency in the present sample had a Cronbach’s α = 0.89.

##### 3.1.2.3 The German psychological need satisfaction in exercise scale

To test for construct validity, we applied the German version of the PNSEG (Rackow et al., 2013), which has been used in several German studies in recent years (e.g., Leisterer and Gramlich, 2021). The questionnaire is composed of three subscales indicating perceived autonomy (e.g., “I feel the way I exercise is an expression of myself”), competence (e.g., “I feel confident in my ability to exercise regularly”) and relatedness (e.g., “I feel connected to the people I interact with while exercising”) in the exercise context. Participants rated items on a 7-point Likert scale ranging from 1 (“do not agree at al”) to 7 (“very strongly agree”). Reliability showed a Cronbach’s *α* for the autonomy scale of 0.79, for the competence scale of 0.87 and for the relatedness scale of 0.86. Participants not involved in any kind of sport did not answer this questionnaire.

##### 3.1.2.4 The international physical activity questionnaire

Physical exercise in this study was assessed via the IPAQ (Booth, 2000). Participants indicated vigorous, moderate and walking activity as well as sedentary periods during the past 7 days. Questions included the number of days in which individuals engaged in the above-mentioned activities for more than 10 min and the length of time they generally spent each time they engaged in these activities. To obtain a weighted score, duration and frequency of each type of activity were multiplied.

##### 3.1.2.5 Demographics

Demographic information was collected as in study 1, with an additional question about the main type of sport (open-ended) practiced. (See the section entitled Demographics in study 1 for a detailed description.)

#### 3.1.3 Data analysis

Due to violations of the normal distribution we used Spearman correlations to analyze construct, criterion, and discriminant validity.

We used SPSS 29 to measure descriptive statistics, scale values, and correlations between the different scales of the respective questionnaires.

### 3.2 Results

#### 3.2.1 Descriptive statistics

Study 2 participants yielded average BPNSS-S scores of 4.55 (*SD* = 1.42) for autonomy, 4.09 (*SD* = 1.39) for competence, and 4.73 (*SD* = 1.26) for relatedness in the sleep domain. The sample showed an average PSQI score of 5.18 (*SD* = 2.75) and an average sum score of the SWLS of 24.42 (*SD* = 5.99). In the exercise domain (PNSEG), the sample attained average scores of 5.47 (*SD* = 1.09) for autonomy, 5.40 (*SD* = 1.09) for competence and 5.18 (*SD* = 1.30) for relatedness. On average, participants exercised 4.25 h (*SD* = 4.31) vigorously and 4.27 h (*SD* = 5.78) moderately per week. Subjects in competitive sports exercised 11.32 h (*SD* = 7.39; national level) and 7.79 h (*SD* = 4.16) vigorously, while subjects in recreational sports trained for 3.87 h (SD = 3.48). Average walking times per week were 7.86 h (*SD* = 12.62). Values can be found in Table 6.

**Table 6:** Means, standard deviations, and correlations among the main study variables.

Measure	*M*	*SD*	1	2	3	4	5	6	7	8	9
Autonomy (Sleep)	4.55	1.43	–								
Competence (Sleep)	4.09	1.39	0.41**	–							
Relatedness (Sleep)	4.73	1.26	0.13*	0.25**	–						
Autonomy (Sport)	5.47	1.09	0.20**	0.14*	0.17**	–					
Competence (Sport)	5.40	1.09	0.12*	0.09	0.08	0.65**	–				
Relatedness (Sport)	5.18	1.30	−0.04	0.04	0.16**	0.39**	0.48**	–			
PSQI Score	5.18	2.75	−0.22**	−0.42**	−0.16**	−0.04	−0.05	0.01	–		
SWLS Score	24.42	5.99	0.20**	0.37**	0.21**	0.10	0.02	0.16**	−0.28**	–	
Vigorous activity (h)	4.25	4.31	−0.05	−0.06	−0.04	0.12*	0.18**	0.14*	0.03	0.04	-

Sample size ranged from *n* = 289 to *n* = 243 due to missing values in the questionnaires. *p < 0.05; **p < 0.01.

In this sample, the BPNSS-S yielded Cronbach’s α = 0.73 for the autonomy scale, α = 0.82 for the competence scale and α = 0.68 for the relatedness scale, which is comparable to the internal consistency measured in study 1. The subscales of the BPNSS-S were positively correlated to each other: autonomy and competence: *r*(288) = 0.41, *p* < 0.001; competence and relatedness: *r*(288) = 0.25, *p* < 0.001; autonomy and relatedness: *r*(288) = 0.13, *p* = 0.014.

We conducted another CFA with the sample from Study 2 to cross-validate the results in both samples. Compared to the CFA in Study 1, some goodness-of-fit indices yielded a lower fit but the results were still in a good to acceptable range. The χ^2^-test was significant with a χ^2^/df ratio of 2.36 which hints to a slightly worse but still acceptable model fit (Schermelleh-Engel et al., 2003). Adjusted CFI (0.97) and TLI (0.95) values indicate excellent to good model fit (Hu and Bentler, 1999). SRMR value (0.05) was below 0.08, indicating a very good model fit. RMSEA with a value of 0.07 points to an acceptable fit (Browne and Cudeck, 1993; Hu and Bentler, 1999). GFI of 0.98 (>0.95) and AGFI close to the threshold (0.94) show a very good to excellent model fit (Hooper et al., 2008). Thus, the analyses suggest that the results represent robust estimates precluding overfitting.

#### 3.2.2 Criterion validity results

Table 6 depicts correlation values between the different scales. Significant correlations between the PSQI and the three subscales of the BPNSS-S showed negative medium to high values implying a relationship between need satisfaction in sleep and sleep quality. All correlations between the SWLS and the BPNSS-S subscales were significant with positive medium to high values indicating a positive relationship between need satisfaction in sleep and well-being.

#### 3.2.3 Construct validity results

The correlation coefficients between the respective subscales of the BPNSS-S and the PNSEG were all positive, and small to medium (Cohen, 1988; Brydges, 2019). The highest correlation was found for the autonomy subscales, followed by the relatedness subscales. Although the correlation between the competence scales was not significant, a statistical trend toward a positive relationship was evident.

#### 3.2.4 Discriminant validity results

As expected, correlation coefficients showed a positive, medium relationship between vigorous activity in the IPAQ and the three subscales of the PNSEG. On the other hand, vigorous activity was not correlated to subscales of the BPNSS-S. The final BPNSS-S can be found in the Supplementary material.

## 4 Discussion

While evidence indicates that there is a relationship between the fulfillment of basic psychological needs and sleep, there is currently no comprehensive instrument that integrates both concepts, nor is there a questionnaire specifically addressing the sleep domain. Consequently, this study sought to develop and validate a German self-report instrument designed to assess the degree of satisfaction of basic psychological needs (autonomy, competence, relatedness) in sleep (BPNSS-S). In a first study we (1) performed two CFAs (prior to and subsequent to item selection) in order to ascertain a factor structure exhibiting strong model fit. In a subsequent study we (2) determined criterion, construct, and discriminant validity.

In the process of validation, we reduced the initial 11 item version of the scale to a 7-item instrument resulting in a shorter, more efficient tool with improved model fit. The good-to-excellent model fit of the final instrument supports the hypothesis that the structural configuration of the questionnaire effectively represents the theoretical framework within the context of self-determination theory. The final version of the BPNSS-S consists of a 2-item autonomy scale, a 2-item competence scale, and a 3-item relatedness scale. We were able to reproduce the reliability values in study 2 and were also able to establish that the final questionnaire showed good agreement with questionnaires on sleep quality, well-being, and need satisfaction in exercise. There was little agreement with vigorous activity indicating the scale’s discriminatory validity to other life domains. This supports the assumption of good criterion, construct, and discriminant validity of the BPNSS-S, and demonstrates support for the 7-item instrument once again.

Compared to participants in study 1, those in study 2 were older with a broader age range, including more females, more students, and more people physically active (participating in recreational sports, at the very least). Nevertheless, participants in both cohorts achieved similar item scores with slightly lower relatedness in the second cohort. Scale reliability as well as correlation between the scales attained comparable values in both studies. These results point to the robustness of the questionnaire across different sample structures.

Both studies showed correlations between the three subscales autonomy, competence, and relatedness; however, the correlation coefficients were not particularly pronounced. This observation is consistent with earlier validation studies which determined that the three needs constitute a cohesive framework and exhibit interrelations, yet remain distinct constructs that cannot be condensed into a singular factor (Rackow et al., 2013; Neubauer and Voss, 2016). While previous research found slightly higher intercorrelations (*r* = 0.34–0.62) than our study, the lower yet statistically significant correlations observed here are deemed to be theoretically acceptable. The differences might be attributable to the varying life domains across studies, potentially affecting the strength of associations between subscales. Additionally, differences in sample characteristics (e.g., age, level of well-being) or in scale formats (e.g., item formulation, number of items, response scale) may have influenced the observed correlations. Overall, participants in both studies scored values above the scale average on all three subscales. These results are comparable with those from the PNSEG validation (Rackow et al., 2013) and might indicate that participants’ sleep need satisfaction was positive, pointing to good health and well-being.

Criterion validity results support the assumption that basic psychological need satisfaction in the sleep domain is related to better sleep quality and increased well-being, which is in line with basic psychological need theory (Ryan and Deci, 2017). Thus, this study contributes to the theoretical understanding of psychological need satisfaction. While there are a plethora of studies on the relationship between need satisfaction and well-being (Ryan et al., 2010; Lataster et al., 2022; Soenens and Vansteenkiste, 2023), research on need satisfaction and sleep quality is still in its infancy (Campbell et al., 2021). The connection between need satisfaction in the sleep domain and sleep quality and well-being that we found in the present study adds to the growing body of evidence suggesting a relationship between the three concepts. Our findings also support the foundations of self-determination theory (SDT; Ryan and Deci, 2000) and highlight the importance of basic psychological need satisfaction for improved sleep quality, optimal functioning, and well-being.

### 4.1 Limitations and future research perspectives

The interpretation of results should consider study limitations. The first limitation appears at the item level. In contrast to the good fit of indicators in the final BPNSS-S, low item values were found for Rel1 and Rel3 in the areas of communality and item residuals, which were also reflected in overall somewhat lower scale reliability. However, we retained these items as scale reliability was in the acceptable range and could not be improved by further item deletion. Moreover, factor loading values and corrected item-total correlation values were in the moderate range. A possible explanation for the low correspondence of items Rel1 and Rel3 with the rest of the questionnaire could be that relatedness is generally more challenging to depict in the sleep domain, as sleep itself is not actively spent with other people. In domain-unspecific questionnaires (“I experience a warm feeling with the people I spend time with.”) and questionnaires on domains in which time is spent with other people (“I feel connected to the people I interact with while we exercise.”), the link seems easier to describe (Rackow et al., 2013; Heissel et al., 2019).

Secondly, a further limitation arises within construct validity. Whereas the relatedness subscale showed a small correlation between the PNSEG and the BPNSS-S and the autonomy subscale showed a medium correlation, the competence subscale showed only a trend. It is notable that the PNSEG was completed only by those who stated that they engaged in recreational sport at the very least (82.5%). In fact, it is possible that the people who indicated involvement in recreational sport do not engage in sport regularly enough to adequately assess the experience of competence in sport. That is, the recreational sports group reported 3.87 h of vigorous activity per week, with 56% reporting less than 3 h. Another reason for differences could be that the experience of competence is perceived differently in different domains such as sport and sleep. While the experience of competence in sport is linked to proper goal setting (Williamson et al., 2022), it may be more related to mastering the implementation of sleep hygiene strategies in the sleep domain (Reid and Dautovich, 2023). Moreover, as sleep is still an underrated topic in most people’s lives, the majority of participants might not have specific sleep goals to work on.

A third limitation could arise from the fact that the questionnaire refers to need satisfaction and not to need frustration. Interestingly, previous domain-specific need questionnaires also only cover the construct of need satisfaction (e.g., Burgueño et al., 2020; La Guardia et al., 2000; Rackow et al., 2013).

Also, the samples consisted primarily of mentally and physically healthy adults who were active in sports with a minimum of a high school diploma. These participants also indicated above-average life satisfaction and a PSQI score at the upper boundary of good sleep quality. Thus, the validation process might have been biased and the tool in its present form might not be generalizable to other populations. Further (validation) studies should consider including less physically active and less well-educated participants. Moreover, it might be insightful to focus on vulnerable groups with possibly lower well-being/higher ill-being (e.g., (sub-)clinical populations), or lower sleep quality (e.g., athletes, night-shift workers).

Furthermore, future validation with other measurement methods should be considered. For example, even though SWLS overlaps with well-being to a large extent, it does not cover all areas (Ryan et al., 2008). The PSQI also only covers part of sleep quality and might be susceptible to the biases of social desirability or introspective abilities. Objective measurements of sleep parameters using polysomnography or actigraphy, for example, could yield additional insights (Fekedulegn et al., 2020). Furthermore, the BPNSS-S was constructed as a one-time questionnaire. As sleep is quite a complex construct, many studies use day-to-day measures (e.g., sleep logs) to gain better insights into participants’ sleep–wake behavior. Future measurements of need satisfaction in sleep would benefit from a single-item diary, similar to the Brief Index of Sleep Control (Grandner et al., 2020). It could also be insightful to look into other sleep–wake related concepts such as chronotype. For examples, late chronotypes’ preferred bedtimes typically run counter to social demands (e.g., working hours), which often lead to “social jetlag” and increased sleep problems (Wittmann et al., 2006). This might also be reflected in a reduced sense of autonomy of late chronotypes.

In addition, open questions remain regarding theoretical foundations (e.g., competence subscale, the direction of links between concepts such as need satisfaction and sleep) that cannot yet be answered. To better understand the construct, in-depth investigations of what constitutes the perception of basic psychological needs during sleep (e.g., through interviews) as well as its predictive value (e.g., predictive validity of the questionnaire) and its consistency over time (e.g., retest reliability) are warranted. Additional insights into people’s sleep might also be afforded by including a need frustration scale for assessment in the sleep domain (Vansteenkiste and Ryan, 2013). And last but not least, translation of the questionnaire to other languages could add to our theoretical understanding by fostering cross-cultural exchange on this research topic.

### 4.2 Applied perspectives

The present study has shown that the BPNSS-S – as a reliable and valid questionnaire – can be used as an anamnesis tool in sleep monitoring. Due to its brevity, the BPNSS-S can serve as an additional indicator to measure satisfaction with sleep quality and sleep schedule, alongside commonly used tools like the PSQI (Buysse et al., 1989). Questionnaire items can also serve as conversation starters with a sleep consultant regarding possible improvements in this area. Feelings of autonomy can be supported by, for example, having more freedom of choice when it comes to one’s own sleep routine. This might include adapting working (and socializing) hours to one’s needs and internal clock (chronotype). The perception of competence can be increased through psychoeducation (e.g., workshops) focusing on the implementation of sleep hygiene strategies. These can also be used to establish neutral contact persons with whom you can talk about your own sleep behavior to enhance relatedness.

### 4.3 Conclusion

This study has evaluated the BPNSS-S as a reliable and valid tool for assessing basic psychological need satisfaction in the sleep domain, serving both researchers and practitioners aiming to understand and improve sleep quality and well-being. Our work lays the foundation for further investigation of sleep need research, with the potential to guide targeted approaches that enhance sleep behavior across various populations.

## Data Availability

The datasets presented in this study can be found in online repositories. The names of the repository/repositories and accession number(s) can be found at: http://doi.org/10.17605/OSF.IO/Q896F.
