# Supplementary material for: Validation of the German basic psychological need satisfaction in sleep scale
Source: Front Psychol. 2025 Sep 17;16:1611551. doi: 10.3389/fpsyg.2025.1611551 (PMC12483872; doi:10.3389/fpsyg.2025.1611551)
Supplement: Supplementary file 1 [file Data_Sheet_1.pdf]

BPNSS-S

Die folgenden Aussagen beziehen sich auf die Situationen, in denen es um Deine Schlafroutine geht (wann gehe ich ins Bett, wann stehe ich auf, wie lange schlafe ich, wo schlafe ich, etc.)

Bitte gib an, in welchem Ausmaß diese Aussagen jeweils auf Dich ganz persönlich zutreffen.

	1 (trifft gar nicht zu)	2	3	4	5	6	7 (trifft voll und ganz zu)
Ich habe das Gefühl, selbst entscheiden zu können, wie lange ich schlafe.	☐	☐	☐	☐	☐	☐	☐
Ich habe das Gefühl, selbst entscheiden zu können, wann ich ins Bett gehe und wann ich aufstehe.	☐	☐	☐	☐	☐	☐	☐
Ich habe den Eindruck, dass ich effektive Strategien habe, um mir genug Schlaf/Erholung zu holen.	☐	☐	☐	☐	☐	☐	☐
Ich bin auf einem guten Weg, meine Schlafziele zu erreichen.	☐	☐	☐	☐	☐	☐	☐
Ich fühle mich mit den Personen verbunden, mit denen ich rede, wenn es um meine Schlaf-/Regenerationszeit geht.[1]	☐	☐	☐	☐	☐	☐	☐
Es gibt Menschen, denen ich vertrauen kann, wenn ich über Schlafprobleme spreche.	☐	☐	☐	☐	☐	☐	☐
Ich fühle mich sehr wohl, wenn ich mit anderen über meine Schlafroutinen rede.	☐	☐	☐	☐	☐	☐	☐

[1]*Solltest Du mit anderen Personen nicht über Deinen Schlaf sprechen, dann stelle dir einfach vor, wie Du Dich fühlen würdest, wenn Du jetzt mit jemandem darüber sprechen würdest.*

© Patricia Frytz, Prof. Dr. Lucie Nikoleizig, Prof. Dr. Anne-Marie Elbe

Auswertung

Pro Item können Werte zwischen 1 (trifft gar nicht zu) bis 7 (trifft voll und ganz zu) erreicht werden. Die Ergebnisse der Subskalen werden aus den Mittelwerten der zugehörigen Items berechnet.

Autonomie: *Mittelwert der Items 1, 2*

Kompetenz: *Mittelwert der Items 3, 4*

Zugehörigkeit: *Mittelwert der Items 5, 6, 7*

© Patricia Frytz, Prof. Dr. Lucie Nikoleizig, Prof. Dr. Anne-Marie Elbe
